# Chloroplast protein translocation pathways and ubiquitin-dependent regulation at a glance

**DOI:** 10.1242/jcs.241125

**Published:** 2023-09-21

**Authors:** Sreedhar Nellaepalli, Anne Sophie Lau, R. Paul Jarvis

**Affiliations:** ^1^ Section of Molecular Plant Biology, Department of Biology, University of Oxford, Oxford OX1 3RB, UK; ^2^ Department of Plant Physiology, Faculty of Biology, University of Kaiserslautern, 67663 Kaiserslautern, Germany

**Keywords:** Chloroplast, Plastid, Protein degradation, Protein import, Protein translocation, Ubiquitin

## Abstract

Chloroplasts conduct photosynthesis and numerous metabolic and signalling processes that enable plant growth and development. Most of the ∼3000 proteins in chloroplasts are nucleus encoded and must be imported from the cytosol. Thus, the protein import machinery of the organelle (the TOC-TIC apparatus) is of fundamental importance for chloroplast biogenesis and operation. Cytosolic factors target chloroplast precursor proteins to the TOC-TIC apparatus, which drives protein import across the envelope membranes into the organelle, before various internal systems mediate downstream routing to different suborganellar compartments. The protein import system is proteolytically regulated by the ubiquitin-proteasome system (UPS), enabling centralized control over the organellar proteome. In addition, the UPS targets a range of chloroplast proteins directly. In this Cell Science at a Glance article and the accompanying poster, we present mechanistic details of these different chloroplast protein targeting and translocation events, and of the UPS systems that regulate chloroplast proteins.

## Teaser

**Summary:** The development and operation of chloroplasts depends on the correct targeting, translocation, assembly and degradation of thousands of different chloroplast proteins. An overview of these diverse proteostatic processes is presented.

## Introduction

Photosynthetic eukaryotes acquired the power to conduct photosynthesis more than a billion years ago, through an endosymbiotic relationship involving engulfed photosynthetic cyanobacteria (Archibald, 2009). Ultimately, the endosymbionts emerged as chloroplasts (plastids), which are semiautonomous organelles in algae and plants and largely responsible for global carbon fixation (Zimorski et al., 2014). Endosymbiosis was accompanied by massive gene transfer from the endosymbiont to the host cell nucleus. In present-day chloroplasts, the organellar genome retains only ∼100 genes, yet the organelles retain the power to conduct photosynthesis and other metabolic processes by harbouring thousands of nucleus-encoded cytosolically synthesized proteins. Sophisticated targeting mechanisms mediate the directional flow of new chloroplast proteins from cytosolic ribosomes to different chloroplast subcompartments (Jarvis and López-Juez, 2013). These mechanisms involve: (1) cytosolic factors that act as molecular chaperones to target newly synthesized proteins to the chloroplast surface; (2) protein translocation complexes in the chloroplast envelope membranes that import proteins into the organelle [the translocons at the outer chloroplast membrane (TOC) and translocons at the inner chloroplast membrane (TIC); collectively TOC-TIC]; and (3) internal sorting systems that distribute new proteins from the translocons to various internal compartments (see poster) (Li and Chiu, 2010; Paila et al., 2015; Shi and Theg, 2013a; Sun and Jarvis, 2023; Thomson et al., 2020).

In addition to photosynthesis and other metabolic processes, chloroplasts play crucial roles in plant development and environmental adaptation, which are dependent on the remodelling of the organellar proteome. Prokaryotic-type proteolytic systems involving several proteases critically influence chloroplast functions, for example by implementing protein degradation to repair and remodel chloroplast bioenergetics (Nishimura et al., 2016). However, a role in chloroplasts for the preeminent eukaryotic-type proteolytic system – the ubiquitin-proteasome system (UPS), which mediates protein degradation in nucleocytosolic compartments and organelles, such as the endoplasmic reticulum (ER) and mitochondria – was not fully appreciated until recently. In the past decade, two breakthrough discoveries revealed that the UPS regulates chloroplast outer envelope membrane (OEM) proteins to control protein import, via a multicomponent system called chloroplast-associated protein degradation (CHLORAD) (Ling et al., 2019, 2012). More recently, the UPS has been shown to also act on proteins in internal chloroplast compartments, including the stroma and thylakoids (Li et al., 2022; Sun et al., 2022). Activity of the UPS in chloroplasts is important during developmental transitions when plastids change type, and under stress conditions (Li et al., 2022; Ling and Jarvis, 2015).

**Figure:**
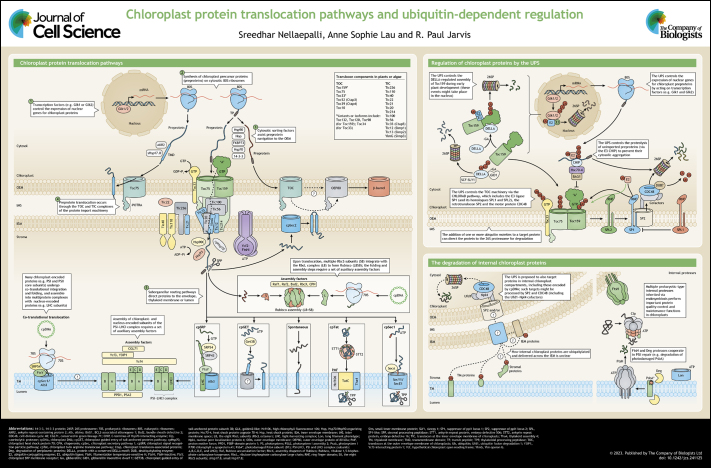
See Supplementary information for a high-resolution version of the poster.

In this Cell Science at a Glance article and on the accompanying poster, we summarize mechanistic aspects of the different chloroplast protein targeting systems, and of the different UPS systems that regulate chloroplast biogenesis and homeostasis.

## Chloroplast protein targeting

### Protein targeting to the chloroplast surface

Numerous nucleus-encoded proteins are required for the establishment of photosynthesis and chloroplast development. Typically, chloroplast proteins are synthesized on cytosolic ribosomes in precursor (preprotein) form, each one with an N-terminal extension called a transit peptide (TP). The TP comprises ∼25–100 residues and acts as a ‘postal address’ for correct delivery to the organelle (Lee and Hwang, 2021).

The TP provides binding motifs for various factors along the protein targeting pathway, ensuring directional transport from the cytosol to the chloroplast stroma (Flores-Pérez and Jarvis, 2013). Cytosolic factors act as molecular chaperones to keep preproteins in an import-competent state, by preventing their folding, aggregation or degradation, and to facilitate navigation from the ribosomes to the chloroplast surface. The cytosolic targeting machinery can include heat-shock protein (Hsp) 90, Hsp70/90-organising protein (Hop), immunophilin FKBP73, Hsp70 and an undefined 14-3-3 protein, although the exact roles of individual components are unclear (Fellerer et al., 2011; May and Soll, 2000). Upon arrival at the chloroplast surface, the membrane-embedded TOC-TIC apparatus mediates protein import, as detailed below, with energy for protein translocation provided by ATP-driven motor complexes at the inner envelope membrane (IEM) (see poster).

Unlike most other chloroplast proteins, OEM-resident proteins with simple α-helical transmembrane domains (such as Toc33 and Toc34) do not possess TPs. For such proteins, targeting information lies in their transmembrane domains, and the cytosolic targeting machinery can include ankyrin repeat-containing protein 2 (AKR2) and small Hsp17.8 (sHsp17.8) (Bae et al., 2008; Kim et al., 2011). Biochemical evidence indicates that AKR2 and sHsp17.8 can deliver newly synthesized OEM proteins to the main import channel (Toc75), although mechanistic details are lacking (Bae et al., 2008; Kim et al., 2011; Tu et al., 2004). Concerning the biogenesis of more complex β-barrel OEM proteins, the outer envelope protein OEP80 (also called Toc75-V; an Omp85 homologue) plays an essential role (Day et al., 2019). Indeed, OEP80 has been proposed to be functionally analogous to β-barrel assembly machinery A (BamA) and sorting and assembly machinery 50 (Sam50), which mediate β-barrel biogenesis in bacteria and mitochondria, respectively (Huang et al., 2011).

### Preprotein translocation across the envelope by TOC, TIC and ATP-driven motors

#### TOC complex

The TOC is a multiprotein translocon in the OEM. It mediates the initial recognition of preproteins at the chloroplast surface and acts as an entry gate for protein import by translocating preproteins across the OEM to the TIC complex (Jarvis and López-Juez, 2013). It consists of three core components: two preprotein receptor GTPases, Toc159 and Toc33, and a channel-forming β-barrel, Toc75 (Bauer et al., 2000; Ertel et al., 2005; Jarvis et al., 1998) (see poster).

The two receptors possess related GTPase domains that face the cytosol; they recognize preproteins by binding to their TP, directing them towards Toc75. Toc159 is structurally complex as it possesses an N-terminal intrinsically-disordered acidic (A) domain (which might contribute to preprotein recognition specificity) and a large C-terminal membrane-embedded domain (predicted to form a 14-stranded β-barrel by AlphaFold), either side of its GTPase domain (Bauer et al., 2000; Chen et al., 2000; Jumper et al., 2021; Kubis et al., 2004). In the model plant *Arabidopsis thaliana*, Toc159 exists in four isoforms, Toc159, Toc132, Toc120 and Toc90, whereas Toc33 exists in two isoforms, Toc33 and Toc34. These isoforms have different substrate specificities for photosynthetic and non-photosynthetic preproteins, enabling a tight regulation of import to control the proteome and functions of the organelle (Jarvis and López-Juez, 2013). Plant mutants for these TOC components are well studied and exhibit moderate-to-lethal phenotypes (Baldwin et al., 2005; Bauer et al., 2000; Jarvis et al., 1998; Kubis et al., 2004).

The Toc75 protein is a member of the Omp85 superfamily, which also includes β-barrels in gram-negative bacteria and mitochondria, and it has long been recognized as the key determinant of import channel formation. Its β-barrel is predicted to have 16 β-strands, and it forms a central pore with a diameter of 14–26 Å based on electrophysiology measurements (Hinnah et al., 2002). In addition to the β-barrel domain, Toc75 possesses a soluble N-terminal polypeptide transport-associated (POTRA) domain facing the intermembrane space (IMS) (Baldwin et al., 2005; O'Neil et al., 2017). The POTRA domain provides a binding site for preproteins as they emerge from the pore, probably providing a chaperone-like activity to prevent misfolding as preproteins pass through the IMS (Paila et al., 2016).

Until recently, empirical structural information on TOC was limited to that of the cytosolic domains of bacterially expressed Toc33 and Toc34 and of the Toc75 POTRA domain at 2.0-2.5 Å resolution, as well as low-resolution imaging of the complex (Koenig et al., 2008; O'Neil et al., 2017; Schleiff et al., 2003; Sun et al., 2002). Now, cryo-EM structures at 2.5-2.8 Å resolution of TOC-TIC supercomplexes from the green alga *Chlamydomonas reinhardtii* are available (Jin et al., 2022; Liu et al., 2023) (see Box 1 and poster for details).

**Box 1. Structure of the green algal TOC-TIC translocon complex**

**Components and their evolutionary conservation**

Recently reported cryo-EM structures of green algal TOC-TIC supercomplexes isolated from *C. reinhardtii* (Jin et al., 2022; Liu et al., 2023) include well-conserved TOC components (i.e. the Toc159-type protein Toc90, Toc75 and Toc34) and TIC components [Tic20, Tic12 (also known as Simp1), Tic35 and YlmG (also known as Simp3)], as well as some green alga-specific TOC and TIC components as follows: Toc52 (Ctap3), Toc39 (Ctap4), Toc10, and Tic13 (Simp2). In addition, the TIC-linked IMS scaffold consists of Tic214, Tic100 and Tic56, which are generally conserved in the green-lineage but absent in grasses (Poaceae).

**Structural details**

The structures show that the C-terminal membrane domain of Toc90 forms a 14-stranded β-barrel (Jin et al., 2022; Liu et al., 2023). Surprisingly, the β-domains of Toc90 and Toc75 form a hybrid channel with an adaptive pore diameter of 11–22 Å. It is speculated that this assembly of Toc75 with Toc90 was derived through the adaptation of substrate-bound BamA complexes in the original endosymbiont. Interestingly, the green algal TOC complex contained three additional, uncharacterized components, called Toc39, Toc52 and Toc10. The Toc39 protein forms a second β-barrel channel in the OEM, whereas Toc52 possesses an N-terminal transmembrane domain and a large IMS domain, and Toc10 presents many charged residues inside the hybrid channel. However, further analysis is needed to reveal the functions of these components in protein import.

The Tic214 protein is the only chloroplast-encoded translocon subunit, and it is anchored in the IEM by a C-terminal membrane domain and is physically connected with the TOC channel via its large IMS domain. Puzzlingly, the Tic214 IMS domain co-folds heavily with Tic100 and Tic56, as well as the IMS domains of Tic35 and Toc52, producing a highly complex arrangement in the IMS. Together, Tic20 and YlmG might constitute a translocation pathway in the TIC complex (Jin et al., 2022), while it has also been proposed that multiple components cooperate to form different exit channels leading to the intermembrane space and stroma (Liu et al., 2023). However, the structural models are insufficient to fully elucidate the import pathways.

#### TIC complex

Unlike that of the TOC complex, the composition of the TIC machinery has remained uncertain, perhaps due to its complex role in receiving proteins exiting the TOC and translocating them across the IEM, as well as in coordinating the final steps of protein import with ATP-driven motor systems (see poster). Early studies identified Tic110 and Tic40 as TIC components (Chou et al., 2003; Inaba et al., 2005), whereas more recent work has shown that Tic214, Tic100, Tic56, Tic20 and Tic12 assemble as a 1-MDa complex in green algae and plants, with the exception of grasses where alternative components might operate (de Vries et al., 2015; Kikuchi et al., 2013; Köhler et al., 2015; Zhao et al., 2022). However, whether and how these distinct systems cooperate during protein import has remained elusive. In plants, knockout mutations affecting the 1-MDa complex components have very severe phenotypes characterized by destabilization of the complex (Kikuchi et al., 2013), whereas partial loss of Tic100 function caused defects in protein import and chloroplast biogenesis (Loudya et al., 2022). Conditional repression of Tic214 in *Chlamydomonas reinhardtii* impairs chloroplast protein import with effects on chloroplast ribosome biogenesis, protein folding and stress responses (Ramundo et al., 2020) (see Box 1 for a detailed description of the algal TIC complex).

In plants, Tic236, which is a homologue of the bacterial inner membrane protein TamB, and Tic22, a ubiquitous plastid IMS protein, were proposed to functionally connect the TOC and TIC complexes (Chen et al., 2018; Kouranov et al., 1998). However, these components are missing in the recent biochemical and structural datasets generated in algae, suggesting that there could be fundamental differences in the import machineries of these evolutionarily distinct groups (Jin et al., 2022; Liu et al., 2023; Ramundo et al., 2020). It is also possible that some components interact transiently and act only to assist the assembly of TOC-TIC complexes.

#### Motor complexes for protein translocation

Energy is required to translocate preproteins across the envelope and into the stroma. However, there is uncertainty regarding what proteins operate in this process, as multiple import motor systems have been proposed. For many years, one or more stromal chaperones (namely, cpHsp70, Hsp90C and Hsp93) were proposed to act in the import motor, following their recruitment to the IEM by TIC components, including Tic110 and Tic40 (Jarvis and López-Juez, 2013; Li et al., 2020; Shi and Theg, 2013b). However, a 2-MDa AAA-ATPase complex consisting of chloroplast-encoded Ycf2, five FtsH-like proteins (FtsHi1, -2, -4, -5 and FtsH12) and an NAD-malate dehydrogenase subunit was recently identified as associating with the 1 MDa TIC complex and was proposed to fulfil motor functions (Kikuchi et al., 2018; Xing et al., 2022). Thus, different motor systems with different substrate specificities or properties are perhaps required to meet all of the requirements of the organelle (Herrmann, 2018).

### Internal sorting machineries

Upon arrival in the stroma, the TP is proteolytically removed by the stromal processing peptidase (SPP) (Richter and Lamppa, 2003). Then, the imported protein can assume its final folded conformation, or be delivered to one of several suborganellar destinations. Pathways that target proteins to the thylakoids and IEM are described briefly below (for more details, refer to Cline and Dabney-Smith, 2008; Sun and Jarvis, 2023; Ziehe et al., 2018).

Multiple pathways deliver proteins to the thylakoids (see poster). Light-harvesting chlorophyll-binding proteins (LHCPs) are targeted to the thylakoid membrane via the chloroplast signal recognition particle (cpSRP) pathway (Rathod et al., 2022; Xu et al., 2021; Ziehe et al., 2018). Two soluble components, cpSRP54 and the chloroplast-specific cpSR43, target LHCPs to the membrane via the receptor cpFtsY and the insertase albino3 (Alb3; this is related to Oxa1p and YidC in mitochondria and bacteria, respectively) (Ziehe et al., 2018). The cpSRP43 protein interacts with a hydrophilic 18-residue L18 motif located between the second and third transmembrane helices of LHCP to enable the unusual post-translational mode of cpSRP action (SRP systems more typically act co-translationally) (DeLille et al., 2000). Other proteins are directed to the thylakoid membrane by a C-terminal transmembrane signal via the chloroplast guided entry of tail-anchored proteins (cpGET) pathway (Anderson et al., 2021; Zhu et al., 2022). This involves the stromal Get3B ATPase and unknown membrane insertase(s) (possibly Alb3 or Alb4). Finally, some membrane proteins are integrated spontaneously without the assistance of known factors (Sun and Jarvis, 2023).

Proteins destined for the thylakoid lumen are delivered by the chloroplast twin-arginine translocase (cpTat) or chloroplast secretory (cpSec1) pathways (Albiniak et al., 2012; New et al., 2018) (see poster). The cpTat pathway involves the core membrane-bound components Tha4, Hcf106 and TatC (homologues of bacterial TatA, TatB and TatC, respectively), as well as the stromal components STT1 and/or STT2, which undergo liquid-liquid phase separation to facilitate protein sorting (Ouyang et al., 2020). Binding of cpTat substrates to STT1 and/or STT2 induces the formation of liquid droplets that are separated from the stromal liquid phase to promote their transport across the stroma; thereafter, interaction of STT1 and/or STT2 with Hcf106 reverses the phase separation to enable translocation across the thylakoid membrane. The cpSec1 pathway involves a soluble ATPase, SecA1, that peripherally associates with the thylakoid membrane in the presence of substrate, and a translocon formed of two integral membrane components, SecE1 and SecY1 (all of these are homologues of bacterial Sec components) (Fernandez, 2018). Thylakoid lumen proteins require an additional targeting signal (the luminal targeting peptide) to engage these pathways, which is removed following translocation by the thylakoidal processing peptidase (TPP) (Shackleton and Robinson, 1991).

Some components of the cpSRP and cpSec1 pathways (cpSRP54, cpFtsY and cpSec1 or Alb3) might additionally cooperate in the co-translational insertion of chloroplast-encoded proteins (Ries et al., 2020), including core subunits of the main thylakoid membrane complexes, photosystem (PS) I, PSII and the cytochrome b_6_f complex. Pigments must also be co-translationally integrated with the nascent apoproteins, and in cyanobacteria, the terminal enzyme of chlorophyll synthesis, chlorophyll synthase, interacts with the YidC insertase and PSII assembly factor Ycf39 (Chidgey et al., 2014); it has been speculated that these physical interactions support co-translational pigment integration.

Proteins destined for the IEM can follow the so-called stop-transfer or post-import (conservative sorting) pathways (Viana et al., 2010). In the former, a preprotein transmembrane domain causes translocation arrest in the TIC complex, leading to its lateral exit into the IEM. In the latter, the preprotein undergoes complete translocation into the stroma before insertion into the IEM in a separate process; this integration step involves a second Sec system called cpSec2, consisting of SecA2, SecE2 and SCY2 (Li et al., 2017).

Upon the arrival of nucleus-encoded proteins at their intended destinations, they often must quickly assemble with chloroplast-encoded proteins to form multiprotein complexes; examples include ribulose bisphosphate carboxylase/oxygenase (Rubisco) in the stroma, the TIC complex in the IEM, and PS complexes in the thylakoid membranes (Rochaix, 2011). Thus, the assembly of such complexes requires the concerted action of the nucleocytosolic and organellar genetic systems, and there are dedicated systems involving multiple chaperones and auxiliary factors that facilitate the assembly processes (see Box 2 for further details on the assembly of Rubisco and PSI).

**Box 2. Assembly of chloroplast multiprotein complexes**

**Assembly of the Rubisco holoenzyme**

Rubisco is a key enzyme for the fixation of atmospheric CO_2_ in photosynthetic organisms. In eukaryotes, Rubisco consists of eight large chloroplast-encoded subunits (RbcL; L8) and eight small nucleus-encoded subunits (RbcS; S8), which form a hexadecameric holoenzyme (L8S8) (see poster). Rubisco biogenesis begins with the expression of *rbcL*, the mRNA for which is stabilized by the binding of a conserved pentatricopeptide repeat protein, MRL1, to its 5′ untranslated region (Johnson et al., 2010). Upon translation, the nascent RbcL polypeptide is folded by a chaperonin (CPN) complex, Cpn60–Cpn20–Cpn10. In plants, several auxiliary factors including RbcX, Bsd2, Raf1 and Raf2 are known to facilitate the oligomerization of RbcL monomers (to form L8) (Aigner et al., 2017). Recently, in *C. reinhardtii*, it has been proposed that L8–Raf1 is the final assembly intermediate complex, which provides a platform for the binding of RbcS following its translocatation into chloroplasts by the TOC-TIC machinery (Wietrzynski et al., 2021). If RbcS becomes limiting, the L8–Raf1 complex exerts negative feedback on RbcL translation to control the biogenesis of Rubisco. However, mechanistic details of the assembly process require further exploration.

**Assembly of the PSI complex**

The assembly of pigment-binding multiprotein complexes in the thylakoid membranes, such as PSI and PSII, is even more complex. The electron transfer reactions of PSI are required for the production of ATP and NADPH, and hence proper complex assembly is crucial. In *C. reinhardtii*, PSI consists of four chloroplast-encoded subunits (PsaA–PsaC and PsaJ) and 19 nucleus-encoded subunits, including ten core PSI subunits (PsaD–PsaI, PsaK, PsaL, PsaN and PsaO) and nine peripheral light-harvesting subunits (LHCA1-9), as well as many cofactors such as chlorophylls, carotenoids, lipids and 4Fe-4S clusters (Suga et al., 2019). Several auxiliary factors including Ycf3, Ycf4, Y3IP1, CGL71, PPD1 and PSA2 play a role in the assembly of PSI (Boudreau et al., 1997; Fristedt et al., 2014; Liu et al., 2012; Nellaepalli et al., 2021, 2018; Ruf et al., 1997) (see poster). It has been proposed that Ycf3 and Y3IP1 facilitate the initial assembly of reaction centre subunits (PsaA and PsaB) (Nellaepalli et al., 2018), and that CGL71 assists the oxygen-sensitive assembly steps, such as integration of 4Fe-4S, by providing protection from molecular oxygen (Nellaepalli et al., 2021), whereas Ycf4 acts as a scaffold to assist the assembly of other PSI and LHCI subunits in the formation of the mature PSI-LHCI complex (Nellaepalli et al., 2018). The PPD1 and PSA2 factors might be required for assembly of reaction centre subunits from the luminal side. However, the exact mechanisms are still unclear.

## Regulation by the ubiquitin-proteasome system and CHLORAD

### The ubiquitin-proteasome system

The UPS targets damaged, misfolded or superfluous proteins for degradation with remarkable specificity. It acts spatiotemporally in diverse eukaryotic systems from yeast to humans, controlling many nucleocytosolic processes (Hershko et al., 1983; Pickart and Rose, 1985). It also targets organelles, such as the endoplasmic reticulum (ER) via ER-associated protein degradation (ERAD), by employing specialized systems to circumvent the associated membrane barriers (Thomson et al., 2020). An enzymatic cascade, involving the E1, E2 and E3 ubiquitylation enzymes, results in the addition of one or more copies of the 76-residue polypeptide ubiquitin to a target protein, directing it to the 26S proteasome in the cytosol or nucleus for degradation. Thereafter, the ubiquitin moieties are recycled by deubiquitylating (DUB) enzymes. Owing to their role in target recognition, E3 ligases are highly diverse and numerous (e.g. in *Arabidopsis*, there are ∼1400 E3s but only ∼40 E2s and two E1s), enabling the system to act specifically on many substrates. Here, we focus on the how UPS controls chloroplast biogenesis, protein import and homeostasis (see poster).

### Regulation of chloroplast biogenesis by nucleocytosolic UPS action

Chloroplast biogenesis is promoted by golden2-like (Glk) transcription factors, which control the expression of nucleus-encoded photosynthesis-related genes (Waters et al., 2009). This must be tightly controlled under stress conditions in order to attenuate photosynthetic activity and limit the potential for photooxidative damage. Recently, it has been shown that stress responses mediated by abscisic acid promote activation of the constitutive photomorphogenic 1 (COP1) E3 ligase; here, COP1 mediates Glk ubiquitylation and degradation, causing reduced expression of photosynthesis and chlorophyll biosynthesis genes, and suppressed chloroplast development, promoting stress resistance (Lee et al., 2021). Activity of Glk is also regulated in response to retrograde plastid signals that sense the developmental or operational status of the organelle (Kakizaki et al., 2009; Tokumaru et al., 2017). Such Glk regulation occurs both at a transcriptional level through plastid-to-nucleus signals mediated via genomes uncoupled 1 (GUN1), and at a post-translational level involving UPS-dependent Glk degradation (see poster).

The UPS also regulates chloroplast biogenesis in conjunction with DELLA proteins, which exert growth-restraining effects during seed germination in a gibberellic acid (GA)-dependent manner (Shanmugabalaji et al., 2018). When levels of the hormone GA are low, DELLA proteins interact with unintegrated Toc159, promoting its UPS-dependent degradation. Thus, Toc159 assembly into TOC complexes is impaired and chloroplast biogenesis is blocked. Under conditions favourable for seed germination, GA accumulates to high levels and forms a complex with the receptor gibberellin insensitive dwarf 1 (GID1), which then interacts with DELLA. This results in ubiquitylation of DELLA by a SCF E3 ligase complex involving the F-box protein sleepy 1 (SLY1), and its degradation (Dill et al., 2004; Hirano et al., 2008). Thus, Toc159 is stabilized and TOC assembly proceeds, enabling chloroplast biogenesis.

The UPS also has a role in controlling preprotein accumulation in the cytosol, which might otherwise result in aggregate formation and cellular damage. Heat shock protein cognate 70-4 (Hsc70-4), the E3 ligase C-terminus of Hsc70-interacting protein (CHIP) and the cofactor BCL2-associated athanogene 1 (BAG1) cooperate in this role (Lee et al., 2016, 2009). The Hsc70-4 chaperone recognizes sequence motifs in TPs to recruit preproteins to CHIP. Such UPS-dependent degradation of cytosolic precursors significantly influences chloroplast biogenesis (Grimmer et al., 2020).

### Regulation of chloroplast protein import

Apart from its roles in nucleocytosolic compartments, the UPS also acts on chloroplast-resident proteins, especially at the OEM where it controls the TOC apparatus and protein import. The relevant proteolytic system has been named chloroplast-associated protein degradation (CHLORAD) (Ling et al., 2019, 2012). Key CHLORAD components were first revealed in a forward-genetic screen for suppressors of the pale-green *plastid protein import1* (*ppi1*) mutant of *Arabidopsis*, which lacks Toc33 (Jarvis et al., 1998). One such suppressor had a defect in the E3 ligase, suppressor of *ppi1* locus 1 (SP1) (Ling et al., 2012). The SP1 protein is located in the OEM, and it possesses two transmembrane domains flanking an IMS domain (for substrate recruitment) and a C-terminal RING domain (for E2 recruitment). It targets TOC components for ubiquitylation and proteasomal degradation, and its identification uncovered a direct connection between the UPS and chloroplast protein homeostasis (Ling et al., 2019, 2012). Suppression of the pale-green phenotype in *sp1 ppi1* mutants was due to reduced TOC degradation by CHLORAD, and consequently improved chloroplast protein import. Recently, CHLORAD has been shown to be controlled by a family of SP1-related E3 ligase genes, including *SPL1* and *SPL2*, enabling nuanced control of the process (Mohd Ali et al., 2023).

The other CHLORAD component identified in the genetic screen was SP2, an Omp85-type β-barrel channel, whereas the cytosolic AAA+ ATPase, cell division cycle 48 (CDC48), was linked to CHLORAD using a proteomics approach (Ling et al., 2019). Although mechanistic details remain to be elucidated, SP2 functions as a retrotranslocation channel for the extraction of ubiquitylated TOC components to the cytosol, and CDC48 uses ATP hydrolysis to generate the force needed to drive such extraction (Ling et al., 2012). Thus, SP2 and CDC48 overcome the physical and energetic barriers to the degradation of TOC by the cytosolic proteasome.

Further investigation revealed that CHLORAD has important functions during environmental responses and development. For example, during abiotic stress, CHLORAD limits the chloroplast import of photosynthetic proteins by depleting the TOC apparatus; this reduces the danger of photooxidative damage owing to overproduction of reactive oxygen species (ROS) by photosynthesis (Ling and Jarvis, 2015). Developmentally, CHLORAD is important when plastids must change type, such as during leaf senescence when chloroplast transform into gerontoplasts, or fruit ripening when chloroplasts transform into chromoplasts (Ling and Jarvis, 2015; Ling et al., 2021). Under these circumstances, CHLORAD helps to reconfigure the TOC machinery so that it is better able to deliver the necessary changes in the organellar proteome. Thus, CHLORAD is responsive to environmental and developmental cues, and has potential as a tool for crop improvement.

### Internal chloroplast protein degradation

Chloroplasts have retained several prokaryotic-type protein-degrading systems from their endosymbiotic origins, which are employed for internal protein homeostasis. These include ATP-dependent proteases, such as caseinolytic protease (Clp), filamentous temperature sensitive H (FtsH) and long filament phenotype (Lon), as well as the ATP-independent protease degradation of periplasmic proteins (Deg) (Adam and Clarke, 2002; Nishimura et al., 2016). The different proteases have different substrates; for example, photodamaged PsbA (PsbA*) is processed by FtsH. For a long time, it was assumed that these prokaryotic-type proteases are exclusively responsible for the degradation of internal chloroplast proteins, even though there have been multiple reports suggesting that ubiquitylation occurs in chloroplasts (Gaspar et al., 1990; Hoffman et al., 1991; Kim et al., 2013; Veierskov and Ferguson, 1991; Wettern et al., 1990; Woodson et al., 2015), and ubiquitylation has been shown to act inside mitochondria (Lavie et al., 2018; Liao et al., 2020).

It was previously believed that CHLORAD action would be restricted to the surface of the organelle (to the OEM), owing to the physical barrier presented by the envelope membranes. However, recent discoveries have revealed that the UPS may reach even to internal chloroplast compartments (Li et al., 2022; Sun et al., 2022) (see poster). Proteomic and biochemical investigations have shown that many internal chloroplast proteins, particularly those related to photosynthesis, are ubiquitylated; this includes proteins in the IEM, stroma and thylakoids, and many others that are encoded by the chloroplast genome. These data, alongside analyses of the physiological consequences of CHLORAD inhibition, imply that ubiquitylation is an important mechanism for the regulation of internal chloroplast proteins. Processing of such proteins involves SP2 and CDC48, although the mechanistic details of ubiquitylation and retrotranslocation (across the IEM) are largely unknown (Sun et al., 2022). A heterodimer of ubiquitin fusion degradation 1 (Ufd1) and nuclear pore localization protein 4 (Npl4) acts as a CDC48 cofactor in the degradation of plastid-encoded proteins upon ROS stress (Li et al., 2022).

## Conclusion and future perspectives

Over the past three decades, major strides have been made towards understanding the import and routing of chloroplast proteins. However, exactly how cytosolic factors target preproteins to the organelle is unclear, and although a well-accepted model for TOC composition exists, a consensus on the TIC apparatus is lacking. Moreover, the protein translocation pathway(s) through TOC and TIC remain unresolved, and another major outstanding challenge is to understand the assembly of the TOC and TIC complexes. Regarding regulation, CHLORAD and the UPS have emerged as important players in chloroplast protein homeostasis, with several key components and physiological functions having been uncovered. However, it is likely that additional components remain undiscovered, while mechanistic investigations are ongoing. Given that CHLORAD plays important roles under environmental stress conditions and developmentally, it might prove useful as a technology for crop improvement.

## Poster

Poster

## Panel 1. Chloroplast protein translocation pathways (I)

Panel 1. Chloroplast protein translocation pathways (I)

## Panel 2. Chloroplast protein translocation pathways (II)

Panel 2. Chloroplast protein translocation pathways (II)

## Panel 3. Regulation of chloroplast proteins by the UPS

Panel 3. Regulation of chloroplast proteins by the UPS

## Panel 4. The degradation of internal chloroplast proteins

Panel 4. The degradation of internal chloroplast proteins

## High-resolution poster and poster panels

A high-resolution version of the poster and individual poster panels are available for downloading at https://journals.biologists.com/jcs/article-lookup/doi/10.1242/jcs.241125#supplementary-data.
