# Supplementary figures and images for: Chloroplast protein translocation pathways and ubiquitin-dependent regulation at a glance
Source: J Cell Sci. 2023 Sep 21;136(18):jcs241125. doi: 10.1242/jcs.241125 (PMC10546890; doi:10.1242/jcs.241125)

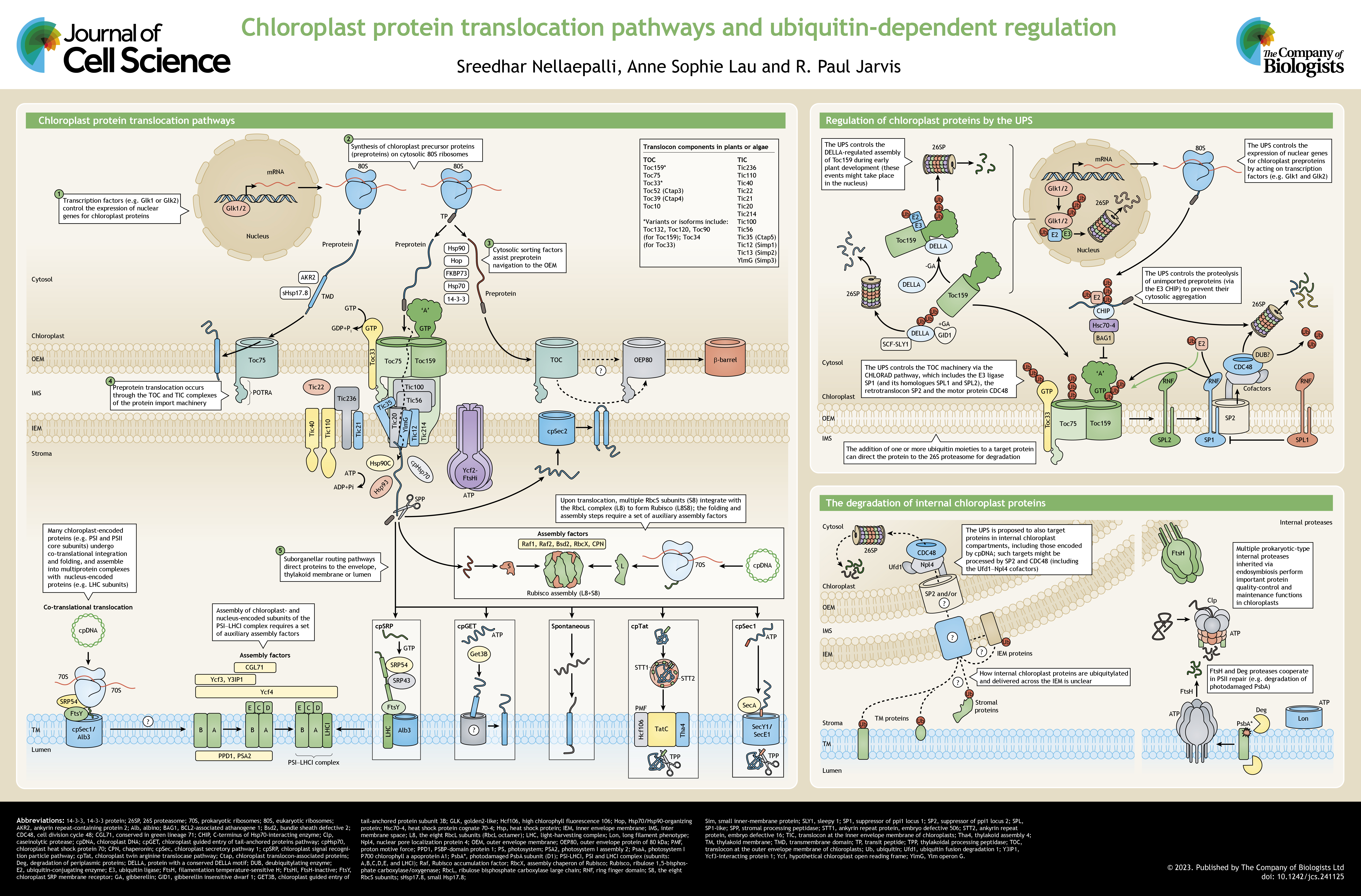

Supplement: Poster [file joces-136-241125-s1.jpg]

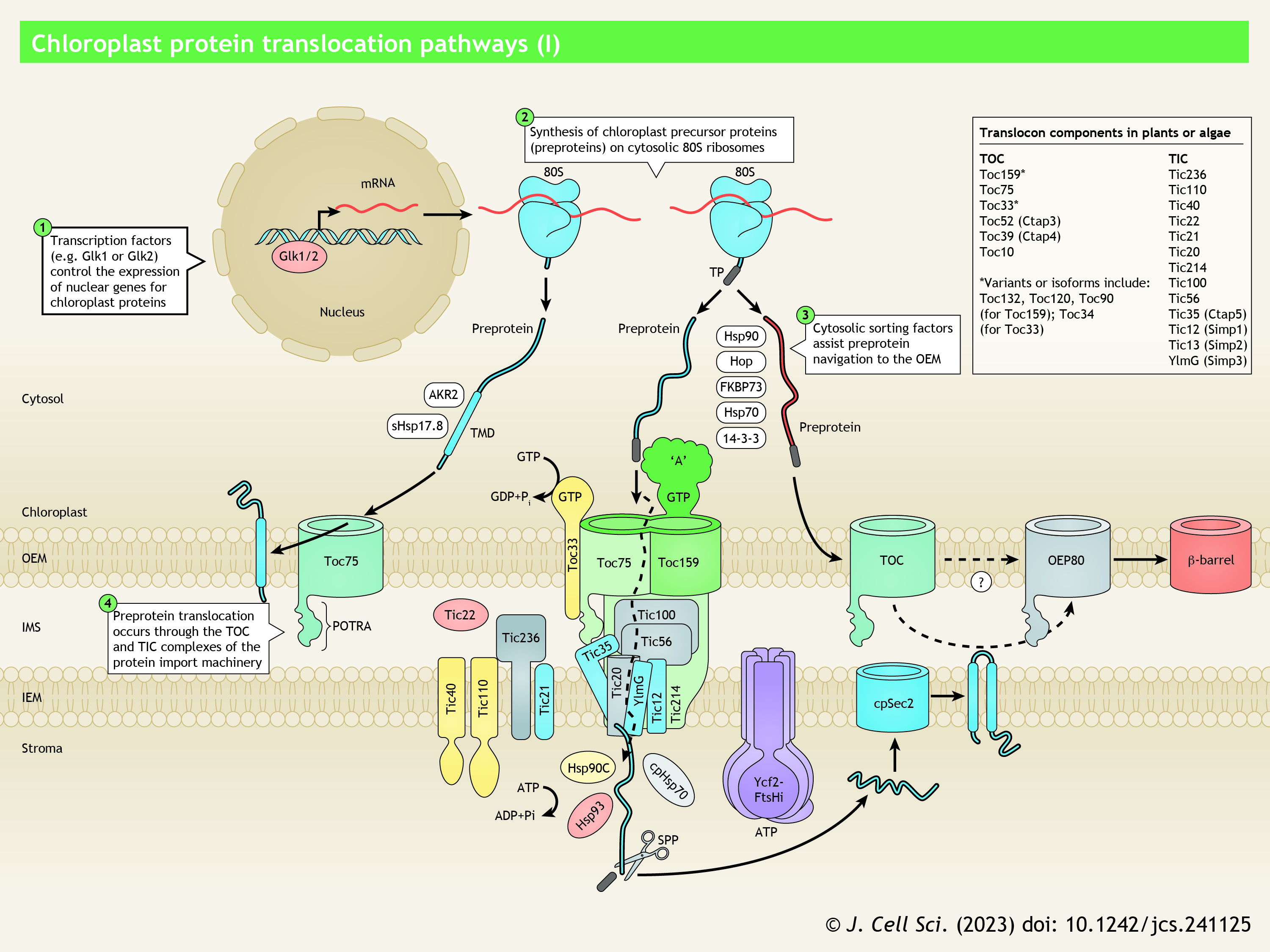

Supplement: Panel 1. Chloroplast protein translocation pathways (I) [file joces-136-241125-s2.jpg]

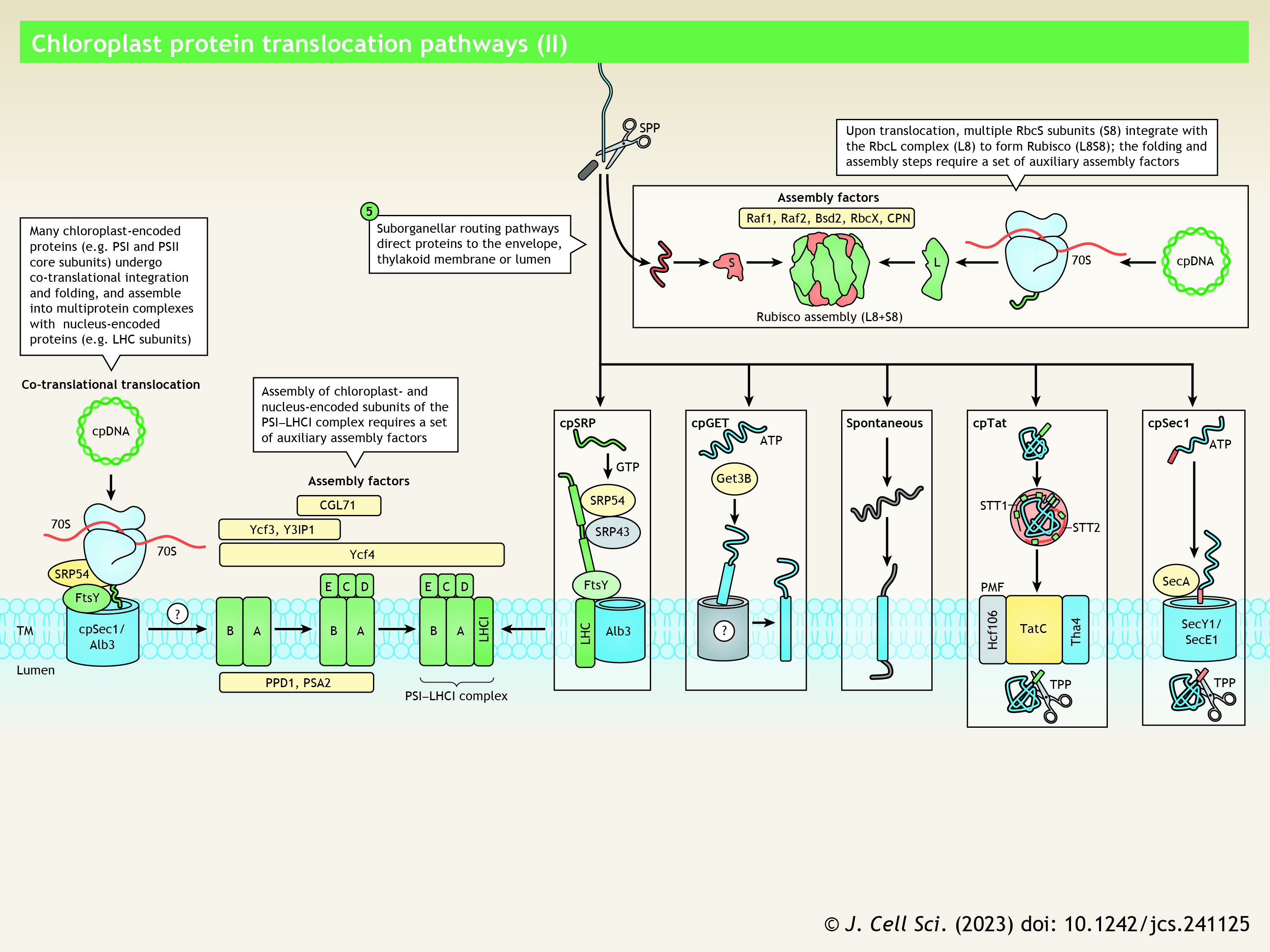

Supplement: Panel 2. Chloroplast protein translocation pathways (II) [file joces-136-241125-s3.jpg]

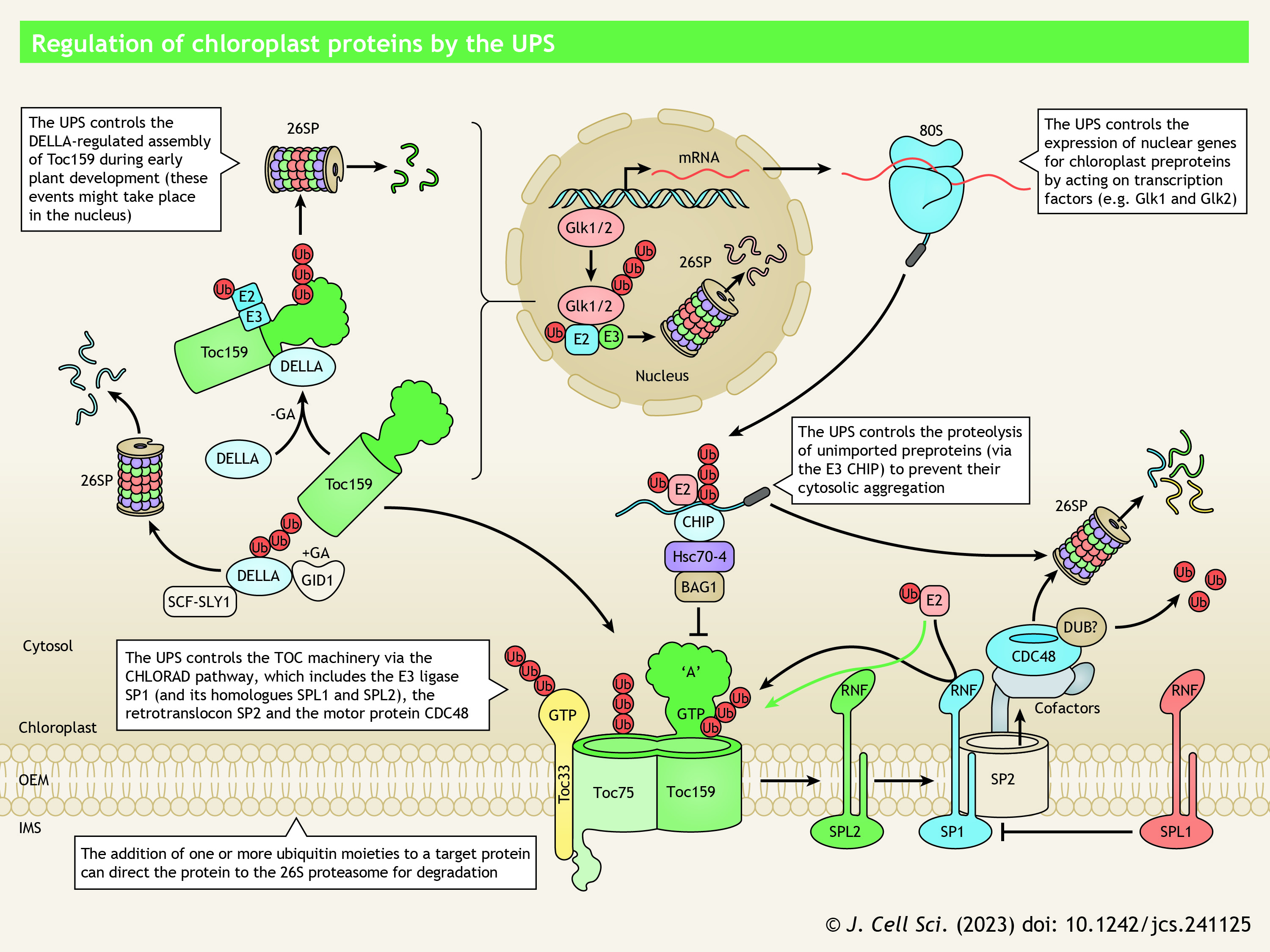

Supplement: Panel 3. Regulation of chloroplast proteins by the UPS [file joces-136-241125-s4.jpg]

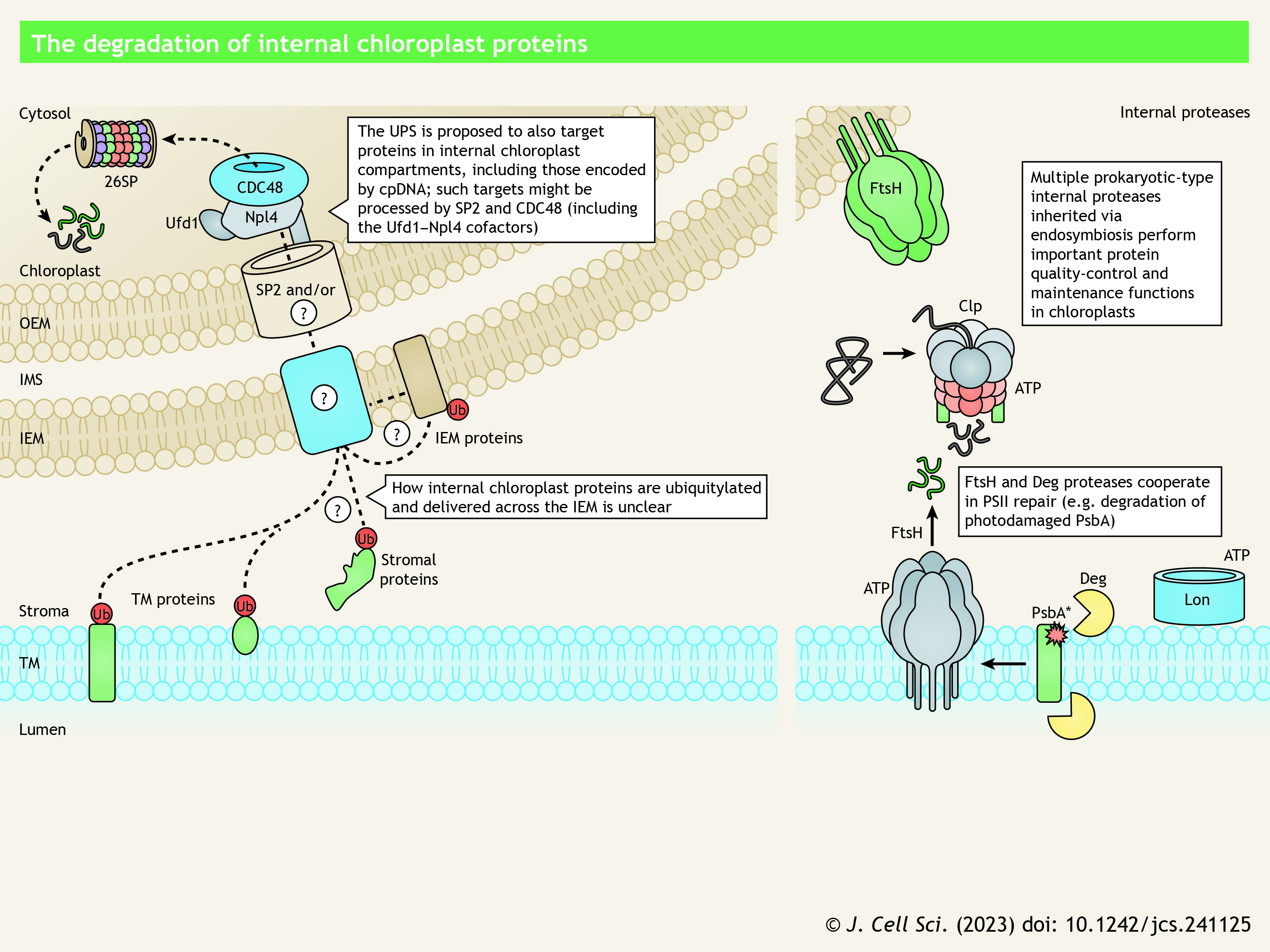

Supplement: Panel 4. The degradation of internal chloroplast proteins [file joces-136-241125-s5.jpg]
